# Oral symptoms and salivary findings in oral lichen planus, oral lichenoid lesions and stomatitis

**DOI:** 10.1186/s12903-017-0393-2

**Published:** 2017-06-29

**Authors:** Kristine Roen Larsen, Jeanne Duus Johansen, Jesper Reibel, Claus Zachariae, Kasper Rosing, Anne Marie Lynge Pedersen

**Affiliations:** 1 Section for Oral Pathology and Oral Medicine, Department of Odontology, Faculty of Health and Medical Sciences, University of Copenhagen, 20 Noerre Allé, DK-2200 Copenhagen N, Denmark; 2 National Allergy Research Centre, Department of Dermatology and Allergy, Gentofte University Hospital, 28 Kildegaardsvej, DK-2900 Hellerup, Denmark; 3 Department of Dermatology and Allergy, Gentofte University Hospital, 28 Kildegaardsvej, DK-2900 Hellerup, Denmark; 4 Section for Community Dentistry, Department of Odontology, Faculty of Health and Medical Sciences, University of Copenhagen, 20 Noerre Allé, DK-2200 Copenhagen N, Denmark

**Keywords:** Oral lichen planus, Lichenoid lesions, Xerostomia, Salivary secretion, Total protein, sIgA

## Abstract

**Background:**

To examine if patients with oral lichen planus, oral lichenoid lesions and generalised stomatitis and concomitant contact allergy have more frequent and severe xerostomia, lower unstimulated and chewing-stimulated saliva and citric-acid-stimulated parotid saliva flow rates, and higher salivary concentration of total protein and sIgA than cases without contact allergy and healthy controls.

**Methods:**

Forty-nine patients (42 women, aged 61.0 ± 10.3 years) and 29 healthy age- and gender-matched subjects underwent a standardised questionnaire on general and oral health, assessment of xerostomia, clinical examination, sialometry, mucosal biopsy and contact allergy testing.

**Results:**

Nineteen patients had oral lichen planus, 19 patients had oral lichenoid lesions and 11 patients had generalised stomatitis. 38.8% had contact allergy. Xerostomia was significantly more common and severe in patients (46.9%) than in healthy controls, whereas the saliva flow rates did not differ. The patients had higher sIgA levels in unstimulated and chewing-stimulated saliva than the healthy controls. The total protein concentration in saliva was lower in the unstimulated saliva samples whereas it was higher in the chewing stimulated saliva samples from patients when compared to healthy controls. The differences were not significant and they were irrespective of the presence of contact allergy.

**Conclusion:**

Xerostomia is prevalent in patients with oral lichen planus, lichenoid lesions and generalised stomatitis, but not associated with salivary gland hypofunction, numbers of systemic diseases or medications, contact allergy, age, or gender. Salivary sIgA levels were higher in patients than in healthy controls, but did not differ between patient groups. The total salivary protein concentration was lower in unstimulated saliva samples and higher in chewing-stimulated saliva samples in patients than in healthy controls, but did not differ between patient groups. Our findings do not aid in the discrimination between OLP and OLL and these conditions with or without contact allergic reactions.

## Background

Oral lichen planus (OLP) is one of the most common oral mucosal lesions affecting 0.5% to 2% of the adult population [1–4]. OLP mainly affects middle-aged and elderly, and is more prevalent in women than in men [5–7]. OLP may present as reticular, erythematous, ulcerative, plaque-like, bullous and papular lesions affecting predominantly the buccal mucosa, gingiva and tongue [3, 5, 6, 8].

The etiology is unknown, but the pathogenesis is believed to involve a T-cell-mediated response. However, the mechanisms triggering the T-cells to enter the oral epithelium and to accumulate in the superficial lamina propria as well the triggering mechanisms behind basal keratinocyte apoptosis are not fully understood, and may involve both antigen-specific and non-specific mechanisms [9]. The diagnosis of OLP is based on fulfillment of clinical and histopathological criteria [10, 11]. Lesions that are clinically and histopathologically similar to OLP may occur as a reaction to certain systemic medications [12] or dental materials [13–15] and are referred to as oral lichenoid lesions (OLL). Also oral hygiene substances, like flavorings, may trigger lichenoid contact sensitivity reactions [16, 17]. At present it may be difficult to distinguish between OLP and OLL. Patients with the erythematous and ulcerative type of OLP often suffer from severe oral mucosal soreness, including burning and itching sensations, particularly in relation to the intake of spicy and acidic food, which may have a negative impact on oral functions as well as the patients’ quality of life and well-being [18]. In addition, we have previously shown that 45% of patients with erythematous and ulcerative OLP also suffer from xerostomia (the subjective feeling of dry mouth) and a sensation of very viscous saliva [18]. These findings have been substantiated by other studies, demonstrating an association between OLP and OLL and xerostomia [19, 20]. OLP and OLL may occur in conditions that are associated with xerostomia and salivary gland hypofunction, including Sjögren’s syndrome, hepatitis C infection, type 1 diabetes, and graft-versus-host disease [21–26]. Patients with these conditions often also display immune-mediated sialoadenitis which may affect the function of the salivary glands. However, it is still debatable whether the complaints of dry mouth and other sicca symptoms should be ascribed the OLP/OLL alone or whether they reflect a pathogenic association to systemic autoimmune diseases in which xerostomia and salivary gland dysfunction are common clinical manifestations. We have previously shown that only 15% of the 45% OLP patients who complained about xerostomia, actually had hyposalivation (i.e., an unstimulated whole saliva flow rate below ≤0.10 ml/min), and this could be related to a daily intake of cardiovascular medications including antihypertensives known to cause xerostomia and/or hyposalivation [18, 27, 28]. A study on salivary gland function and morphology showed that 87% of 39 patients with OLP had a low or very low unstimulated whole saliva flow rate, whereas the chewing-stimulated whole saliva flow rate, the buffering capacity and the salivary pH level were normal. A labial salivary gland biopsy was performed in 15 patients, revealing lymphocytic infiltration (in 80%), acinar atrophy (in 93%), fibrosis, fatty degeneration, or ductal changes. However, although they displayed comparable signs of Sjögren’s syndrome, none fulfilled the classification criteria [29]. Another study on 100 patients with OLP found no associations between OLP and low unstimulated and citric acid stimulated whole saliva flow rates [30].

Xerostomia denotes the subjective feeling of oral dryness, and it is often caused by salivary gland hypofunction, but may occur in the presence of an apparently normal salivary secretion, indicating that not only quantity but also quality of saliva is of importance to the feeling of oral comfort [31, 32]. Accordingly, changes in saliva composition may affect mucosal adhesiveness and lubrication [33]. Moreover, salivary secretion can be reduced as much as 50% of a person’s normal whole saliva flow rate, before the sensation of oral dryness occurs [34]. Regarding changes in saliva composition, it has recently been reported that the total protein concentration was higher in patients with OLP than in healthy control subjects [35]. However, Gandara and co-workers found no differences between patients with OLP and healthy controls in terms of saliva flow rates, salivary proteins and electrolytes [36]. Moreover, it has been shown that patients with OLP and OLL have higher levels of salivary immunoglobulins IgA and IgG than healthy control subjects [35, 37, 38]. Secretory immunoglobin A (sIgA) and immunoglobulin G (IgG) are the two major classes of antibodies in human saliva comprising, 90–98% and 1–10%, respectively [39]. It has been suggested that both serum and salivary immunoglobulins, and particularly sIgA and IgG, play an important role in the pathogenesis of OLP, and it has also been hypothesised that the levels of sIgA and IgG could be useful in discriminating the OLP from OLL [38].

The aim of this cross-sectional study was to examine whether xerostomia, the degree of xerostomia, the unstimulated and chewing-stimulated whole saliva and citric-acid-stimulated parotid saliva flow rates, and the salivary concentration of total protein and sIgA could be used as diagnostic tools in discriminating between patients with OLP, OLL and generalised stomatitis with and without contact allergy.

We hypothesised that patients with OLP, OLL and generalised stomatitis and concomitant contact allergies have a higher frequency of xerostomia, more severe xerostomia, lower unstimulated and chewing-stimulated whole saliva and citric-acid-stimulated parotid saliva flow rates, and higher salivary concentration of total protein and sIgA than patients with OLP, OLL and stomatitis but *without* contact allergies; and healthy age- and gender-matched control subjects.

## Methods

The study was approved by the Regional Ethics Committee, Copenhagen, Denmark (no. H-3-2013-033, March 26^th^ 2013) and conducted according to the Declaration of Helsinki. The participants were both informed by letter and orally and all gave written consent prior to inclusion.

### Study participants

One hundred and thirty-four consecutive patients referred to the Clinic for Oral Medicine, Department of Odontology, Faculty of Health and Medical Sciences, University of Copenhagen, due to symptoms and signs of oral mucosal diseases were screened for inclusion in the study. Fifty-two Caucasian patients were eligible for inclusion of whom 49 (94.2%) completed the study. The remaining patients were excluded due to other diagnoses than OLP, OLL and stomatitis, and in cases where intake of medication was suspected as the eliciting cause of their mucosal lesions. Twenty-nine healthy age- and gender-matched subjects were included via the Danish website for study subjects (www.forsoegsperson.dk). The exclusion criteria for this group were past or current history of systemic and oral diseases as well as intake of medication. As it proved difficult to recruit healthy non-medicated control subjects at the age above 65 years, 4 persons taking antihypertensives (but otherwise healthy) were matched to the patients with regard to gender, age and type of antihypertensive agent.

An oral smear was taken from all study participants prior to inclusion in order to exclude oral candidiasis as it may mimic other mucosal lesions, e.g. erythematous OLP. A superimposed fungal infection may also masquerade the pattern of OLP lesions and be the cause of oral symptoms. The smear was stained with *Periodic Acid Schiff* and evaluated cytologically for presence of yeast hyphae and spores. Eleven patients, but none of the healthy subjects, had an oral candidiasis. They were treated with nystatin for 4 weeks, prior to inclusion. The treatment had no impact on their oral symptoms, but according to a repeated smear, the hyphae and spores were eliminated.

All patients had oral symptoms including stinging and burning sensations, and were diagnosed with OLP, OLL or generalised stomatitis. The patients with OLP and OLL were clinically characterised by various combinations of reticular, erythematous, ulcerative and plaque-like mucosal changes and the diagnosis was confirmed by a biopsy and histopathological examination according to van der Meij and van der Waal [11]. However, this method to discriminate between OLP and OLL still needs to be validated in larger scale studies. The patients diagnosed with stomatitis were characterised by having a more diffuse, widespread oral mucosal erythema. The patients with OLP and OLL were pooled in one group as a strict distinction between the two entities was difficult to make.

All participants underwent a mucosal biopsy at the Department of Odontology, University of Copenhagen, and were referred for patch testing for contact allergy at the Department of Dermatology and Allergy, Gentofte University Hospital. Patch testing to the European baseline series, a toothpaste series and a dental material series were done according to the European Society of Contact Dermatitis (ESCD) guidelines [40]. Moreover, all participants had a check of the serum levels of thyroid stimulating hormone (TSH). They also underwent an interview including standardised questions regarding present and past systemic diseases, including allergies, daily intake of medication, habits regarding alcohol consumption, tobacco smoking and oral hygiene. Data on smoking habits was used to categorize participants as never smokers, former smokers and current smokers. Data on alcohol consumption was used to pool the participants in groups of never consuming alcohol, occasionally or daily consumption of alcohol.

### Assessment of xerostomia

Apart from being questioned about symptoms of the oral mucosa like stinging, burning and roughness, the participants were also asked whether they had taste disturbances and the character of their disturbances in taste perception. Moreover, the participants were asked whether they had symptoms of dry mouth, and if present they were also asked to score the severity of xerostomia using a categorized questionnaire based on Beck’s inventory scale on how questionnaires should be designed in order to obtain valid responses [41] including four degrees of severity (scores 0–3) [42]. The assessment also included potential affection of oropharyngeal functions and diurnal variation in the sensation of oral dryness (Table 1).

**Table 1:** Questionnaire regarding xerostomia and scoring of the severity of xerostomia by patients and healthy controls [41]

Category	Patients *n* = 49	Healthy controls *n* = 29
No feeling of dry mouth (score 0)	53. 1%	79.3%
Slight feeling of dry mouth (score 1)	26.5%	20.7%
Severe feeling of dry mouth (score 2)	8.2%	0%
Annoying feeling of dry mouth making speech difficult (score 3)	12.2%	0%
Difficulty swallowing due to xerostomia	12.2%	0%
Difficulty chewing due to xerostomia	10.2%	0%
Difficulty in eating dry food substances due to xerostomia	32.7%	0%
Taste perception different from normal	28.6%	0%
-increased taste perception	4.1%	0%
-decreased taste perception	18.4%	0%
-no taste perception	4.1%	0%
-altered taste perception (dysgeusia)	14.3%	0%
Nocturnal xerostomia	24.5%	13.8% ^a^
Morning xerostomia	36.7%	24.1%^a^
Daytime xerostomia	22.4%	3.4%
Evening xerostomia	10.2%	0%
Xerostomia at all times of the day	10.2%	0%
Waking up due to xerostomia	34.7%	17.2%^a^
Duration of xerostomia, years	2,88	2

Number of participants reporting symptoms are given in percentage. ^a^Reported to be caused by periodic snoring, in periods without snoring they did not have xerostomia

### Measurements of whole saliva and parotid saliva flow rates

One examiner (KRL) conducted the collection of the whole saliva and the parotid saliva. In brief, the participants were asked to refrain from drinking, eating, chewing chewing-gum/pastilles, tooth brushing and smoking for one hour prior to the salivary measurement. The whole saliva samples were collected using the draining method [43]. Unstimulated and paraffin-chewing-stimulated whole saliva flow rates (UWS and SWS, respectively) were sampled over a 15-min and a 5 min-period, respectively. The citric acid stimulated parotid saliva (SPS) was sampled over a 5-min period [43]. To minimize the influence of the circadian cycle on the composition and flow rate of saliva, all procedures were performed in the same order between 9.00 am and 11.00 am every time. The saliva samples were immediately weighed so that the flow rates could be established and then portioned in 1.5 ml Eppendorf tubes, centrifuged at 10.000 g for 20 min at 4^o^ C, portioned and frozen at -80^o^ C until further analysis. UWS values of ≤0.10 ml/min and SWS values of ≤0.70 ml/min were designated hyposalivation [42]. Patients with UWS flow rates below 0.20 ml/min were designated as low secretors [44].

### Clinical oral examination

One examiner (KRL) conducted the oral clinical examination, calibrated against an experienced clinical examiner (AMLP). The localisation, size and colour of the oral lesions were registered and clinical photos were taken. Also the clinical signs of mucosal dryness were registered.

### Analyses of salivary total protein and sIgA

The total protein concentration (μg/ml) in unstimulated and chewing-stimulated whole saliva was determined using a colorimetric assay [45]. An indirect enzyme immunoassay kit was used to determine sIgA in the whole saliva samples according to the protocol supplied by Salimetrics® (Salimetrics, PA, USA). The out-put of IgA (μg/min) was determined by each concentration was multiplied with the respective salivary flow rate (ml/min).

### Statistics

The statistical analyses were performed using SPSS Version 22 (IBM). Fisher’s test was used for analysis of distributions between the patient group, including those with or without a concomitant contact allergy, and the healthy control group. As the saliva flow rates (UWS, SWS and SPS) were not normally distributed in the patient group, the Mann–Whitney *U* test was used. A *t*-test was used after fulfilling the Shapiro-Wilk’s and Levene’s tests, when comparing the data from the patients with OLL with and without a concomitant contact allergy. The Mann–Whitney *U* test was used for comparisons in salivary total protein and sIgA between the patients and the healthy control subjects, and between patients with OLL with and without a concomitant contact allergy. Associations between variables were analysed by the Spearman rank order correlation test. Statistical significance was defined as *p* < 0.05.

## Results

About 85% of the participants were women, and the average age was 61 years (median 65 years, range 32–77 years). Three patients used tobacco on a daily basis and on average they had smoked 21.5 smoking pack years. Four healthy controls used tobacco daily and they had on average smoked 17.3 smoking pack years. Nineteen patients and 14 healthy controls had ceased smoking more than one year prior to the inclusion in the project. Seven patients and eleven healthy controls reported a daily consumption of alcohol. One male patient reported an alcohol consumption that exceeded the 21 units of alcohol per week limit as recommended by the Danish Health Authority. Forty-six (85.7%) patients reported having one or more medical condition/disease (median 2, range 1–12). The most common ones included recurrent herpes labialis (herpes simplex virus, *n* = 20), hypertension (*n* = 11), osteoarthritis (*n* = 12), contact dermatitis (*n* = 9) and asthma (*n* = 7). Furthermore, 6 patients had pollen allergy, 7 had contact allergy to nickel and 5 had allergy to fragrance ingredients. These were all confirmed prior to the inclusion in the project by testing. General information on the participants can be seen in Table 2.

**Table 2:** Clinical characteristics of the patients and healthy controls

	Patients (*n* = 49)	Healthy controls (*n* = 29)
Female:male ratio	42:7	25:4
Age, years	61.0 ± 10.3 (range 31–77)	58.2 ± 12.1 (range 23–73)
Smoking, percentage (smoking pack year)		
-non	55.1% (0)	37.9% (0)
-former	38.8% (21.2)	48.3% (12.8)
-current	6.1% (21.5)	13.8% (17.3)
Alcohol consumption, percentage		
-never	61.2%	17.2%
-occasionally	24.5%	44.8%
-daily	14.3%	37.9%
Chronic medical conditions (no. given in%)		
-herpes labialis	40.8%	41.4%
-hypertension	24.5%	6.9%
-osteoarthritis	24.5%	6.9%
-hypercholesterolaemia	10.2%	6.9%
-allergic rhinitis	12.2%	0%
-asthma	14.3%	3.4%
-contact allergy	38.8%	34.5%

Data are given in mean and SD

Thirty-two (65.3%) patients reported daily intake of prescribed medication (median 2, range 1–10), most commonly antihypertensives. Sixteen (32.6%) patients had a daily intake of more than two different types of medication (i.e., polypharmacy), 7 patients took two types of medication daily and 9 patients one type of medication daily. Three female patients (6%) had hypothyroidism and one female patient had hyperthyroidism. Blood samples analyzed for levels of thyroid stimulating hormone (TSH) revealed that all patients but two had TSH levels within the normal range. In one patient with known hypothyroidism, the level of TSH was elevated, and her medical treatment was consequently regulated. In another patient with known hyperthyroidism, the level of TSH was decreased, and her medical treatment was adjusted accordingly. In none of these two patients, the adjustments in their treatment lead to changes in their oral condition. Three healthy control subjects showed elevated levels of TSH and were referred to additional medical examinations of possible hypothyroidism. There were no associations between age, gender and the number of medical conditions/diseases and the number of medication taken on daily basis.

The diagnosis of OLP was established in 19 patients and additionally 19 patients were diagnosed with OLL. Fifteen patients had the erosive form of OLP at the time of the examination. As an exact distinction between OLP and OLL was difficult to make, we pooled the patients in one group comprising both OLP and OLL. None of the patients were diagnosed with hepatitis C, Sjögren’s syndrome or any other autoimmune disease. The diagnosis of stomatitis was made in 11 patients (22.4%). Nineteen patients (38.8%) and 10 (34.5%) healthy control subjects were diagnosed with contact allergy, primarily to fragrance mix. Contact allergy to aroma substances in oral hygiene products differed significantly between the patients and the healthy control subjects (*p* = 0.02), primarily in patients with OLP and OLL (*p* = 0.01). Spearmint was the most common allergen in the patient group, whereas it was cassia oil in the healthy controls. The substances that the participants primarily displayed positive patch test reaction to are listed in Table 3.

**Table 3:** Substances that the participants primarily displayed positive patch test reaction to

Substances with positive patch test reactions	Patients (*n* = 49)	Healthy controls (*n* = 29)
Perfume mix	7	4
Balsam of Peru	5	1
Colophony	4	0
Spearmint	4	0
Cassai oil	3	1
Carvone	2	0
Cinnemaldehyd	1	0
Nickel	2	4
Mercury	2	0
2-hydroxyethyl-methacrylate	2	0
Methylmethacrylate (MMA)	1	0
Ethylen glycol dimethacrylate (EDGMA)	1	0
Palladium	1	1
Cobalt	1	1

Some participants displayed more than one positive patch test reaction which explains why the total number adds up to more than the number of patients and healthy controls [56]

Table 1 shows the distribution of xerostomia in terms of severity of xerostomia and any diurnal variations in the patient group with OLP/OLL and stomatitis and the healthy control group. Expectedly, the patients had significantly more complaints of xerostomia and more severe xerostomia than the healthy control subjects (*p* < 0.001). However, there were no differences in the frequency of xerostomia between patients with OLP, OLL and stomatitis, and patients with and without a concomitant contact allergy (*p* = 0.30). There were no associations between the presence and severity of xerostomia and age, gender, number of medical conditions/diseases, including allergies, or the number of medications taken on a daily basis. More female patients than male patients reported xerostomia, but the majority of the study population also comprised women. 28.6% of the patients reported that they experienced their taste perception to be different from normal, primarily as a decreased or an altered taste perception.

The salivary flow rates are listed in Table 4. No differences could be found between the patient group and the healthy control group or between the patients with OLP, OLL and generalised stomatitis with and without a concomitant contact allergy regarding the UWS, SWS and SPS flow rates. However, the UWS flow rates tended to be lower in patients with OLP/OLL and concomitant contact allergy than in those without contact allergy (*p* = 0.05). One patient had severely reduced UWS, SWS and SPS with UWS flow rates below 0.10 ml/min and SWS flow rates ≤0.70 ml/min. Three other patients had UWS hyposalivation, whereas 10 patients were characterised as low secretors. Moreover, two patients had SWS flow rates ≤0.70 ml/min, but normal UWS flow rates. One healthy control subject had UWS hyposalivation and 8 were low secretors, but SWS flow rates were in the normal range. There were no associations between the salivary flow rates (UWS, SWS and SPS) and age, gender, number of medical conditions/diseases, the number of medications taken on a daily basis or the prevalence and severity of xerostomia. The patients with UWS hyposalivation had a salivary gland biopsy taken from the minor glands in the mucosa of the lower lip under the suspicion of Sjögren’s syndrome. None of them had focal lymphocytic infiltration in their glandular tissue or serum autoantibodies, excluding Sjögren’s syndrome. The SPS flow rates did not differ between the patients and the healthy controls or between the patients with and without a concomitant contact allergy.

**Table 4:** Unstimulated (UWS), paraffin-chewing-stimulated (SWS) and citric-acid-stimulated parotid (SPS) saliva flow rates (ml/min), total salivary protein concentration (μg/min) and salivary sIgA levels (μg/min) in unstimulated and chewing-stimulated whole saliva

	Patients (*n* = 49)	Healthy controls (*n* = 29)	*p*-value
UWS (ml/min)	0.33 ± 0.21	0.39 ± 0.25	0.33
SWS (ml/min)	1.69 ± 1.03	1.75 ± 0.72	0.25
SPS (ml/min)	0.15 ± 0.16	0.15 ± 0.12	0.56
Total protein concentration (μg/min)			
-UWS	1047.9 ± 633.0	1131.0 ± 640.1	0.86
-SWS	4317.6 ± 2414.6	4090.0 ± 1689.1	0.58
Levels of sIgA (μg/min)			
-UWS	48.6 ± 29.5	47.8 ± 21.79	0.82
-SWS	96.1 ± 51.7	82.4 ± 38.1	0.19

All data are given in mean and SD

The total protein concentration in terms of output (μg/min) in unstimulated and chewing-stimulated whole saliva from patients and healthy control subjects are listed in Table 4. The patients had higher total protein concentrations in unstimulated whole saliva while it was lower in chewing-stimulated whole saliva but the differences were not significant (*p* = 0.58 and *p* = 0.86 respectively), whereas there were no differences in the total salivary protein concentrations when comparing patients with OLL with and without a concomitant contact allergy.

The levels of sIgA in terms of output (μg/min) in both unstimulated and chewing-stimulated whole saliva from patients and healthy control subjects are shown in Table 4. The levels of sIgA were higher in both unstimulated and chewing-stimulated whole saliva in patients than in healthy control subjects but the difference was not significant (*p* = 0.82 and *p* = 0.19 respectively). No difference could be found in the salivary sIgA levels when comparing patients with OLP/OLL with and without a concomitant contact allergy.

## Discussion

The aim of this cross-sectional study was to determine if xerostomia, degree of xerostomia, UWS, SWS and SPS flow rates and levels of salivary protein concentration and sIgA could be used in discriminating between patients with OLP/OLL and generalised stomatitis with or without concomitant allergy and healthy control subjects.

The majority of patients included in this study were females, which reflects the fact that more women than men are referred to medical clinics and that OLP/OLL are more prevalent in women. However, it also explains the high prevalence xerostomia and other symptoms, the high number of medical diseases/medical conditions and number of medications taken on a daily basis in our study. Furthermore, the age of onset of OLP also indicate that gender hormones may be involved in the pathogenesis via immunological and endocrinological changes affecting the oral mucosa and the immunological response and making the mucosa more susceptible to oral diseases like OLP and allergic reactions.

The diagnosis of OLP and OLL was established in 38 patients (77.6%) according to the recommendations of van der Meij and van der Waal [11]. Although the latter seeks to differentiate between OLP and OLL based on clinicopathological criteria, the value of distinction between them is still controversial. The remaining 11 patients were diagnosed with generalised stomatitis. These patients displayed more diffuse reactions varying from barely visible erythema to bright red erythema anywhere in the oral mucosa. Erosions and hyperkeratosis were also seen as previously described [46]. In our study, we did not find a clear distinction between OLP and OLL with respect to the symptoms and the clinicopathological features.

It is well known that OLL may occur as adverse reactions to systemic drugs including angiotensin-converting enzyme (ACE) inhibitors and beta-blockers [12, 47]. In this study, we eliminated cases in which there was a clear temporal association between the intake of medication and the onset of oral symptoms and signs of OLL. In these cases we found that discontinuation of the medication also resulted in a gradual disappearance of the lichenoid reactions.

As expected, xerostomia was reported significantly more often by the patients (46.9%) than healthy control subjects (20.7%). We did not find any differences between the patients with OLP/OLL and generalised stomatitis with and without contact allergies in terms of prevalence and severity of xerostomia. It is well-known that certain systemic diseases and the intake of certain medications as well as the number of diseases and medications are associated with xerostomia [28, 48–51]. In our study, 65.3% of the patients reported daily intake of medication and 32.6% had an intake of more than 2 different agents on a daily basis, which is reflecting the medication intake in the background population. Several of the medications taken by the participants in our study are known to be “xerogenic” [27, 28]. This might explain the difference in the reports of xerostomia between the patients and the healthy controls. However, we did not find significant associations between the number of medical conditions/diseases, the number of medications and the presence and severity of xerostomia, which may reflect the fact that there were relatively few observations in each category of diseases and medications. However, there was tendency towards an association between the intake of antihypertensives and xerostomia. On the other hand, the sensation of dry mouth could also be solely related to OLP or OLL as previously described [18–20]. It is also well-known that women report xerostomia more frequently than men [48] and, as mentioned earlier, the majority of patients included in this study were women. In the healthy control group, 20.7% reported a “slight sensation of dry mouth”, which was related to awakening and “snoring”. Xerostomia is often associated with salivary gland hypofunction, but in our study we did not find any associations between xerostomia, the severity of xerostomia and the salivary flow rates. Thus, it is likely that the inflammatory changes in the oral mucosa due to OLP, OLL and generalised stomatitis lead the sensory disturbances, including sensation of oral dryness [52].

We found no significant differences in UWS, SWS and SPS flow rates between the patients with and without concomitant allergies and the healthy control subjects, although there was a tendency towards lower UWS flow rates in patients with OLP/OLL and a concomitant contact allergy. Our findings are in concordance with several other studies showing that the salivary flow rates in patients diagnosed with OLP do not differ from the salivary flow rates in healthy control subjects [18, 20, 36, 37].

We found no associations between age, gender and the number of diseases and the number of medication taken on daily basis, which can be ascribed to the very narrow variation in age (the median age was 65 years).

We found higher total protein concentrations in chewing-stimulated saliva samples from patients with OLP/OLL and generalised stomatitis compared to those from healthy control subjects whereas the opposite could be seen in the unstimulated saliva samples. These differences were not significant. Changes in saliva composition can contribute to explain sensation of dry mouth. Our findings are in concordance with Gandara et al. [36] and Artico et al. [20], who found no differences in total protein concentration between patients with OLP and healthy controls. We found no difference either in the total salivary protein concentration between patients with OLP/OLL with and without a concomitant contact allergy.

In our study, the levels of salivary sIgA were higher in both unstimulated and chewing-stimulated whole saliva samples from patients than in those from the healthy control subjects, but the difference was not significant. Previous findings on the levels of salivary sIgA concentrations are contradictory [35–38]. SIgA is an important part of the immune defense of mucosal surfaces. Salivary IgA can be used to evaluate the immune state and very high levels can indicate underlying pathology [37, 53, 54]. We found no difference in the salivary sIgA levels when comparing patients with OLP/OLL with and without a concomitant contact allergy.

A recent study showed that thyroid disease was present in 33 of 215 (15.3%) of patients with OLP compared to 12 of 215 (5.2%) in a control group [55]. However, in our study only 4 patients (6.1%) had a thyroid disease and the serum levels of TSH revealed that three of the healthy control might have hypothyroidism.

It is likely that the higher prevalence and more severe xerostomia, the higher salivary concentrations of total protein and levels of sIgA that we find may be ascribed to a higher degree of anxiety, depression and sleep disturbances in the patient group. These speculations are supported by a recent study of Lopez-Jornet et al. [35] showing that patients OLP presented worse psychological profiles and sleep disturbances, and also higher values for sIgA, cortisol, and total proteins than control subjects.

## Conclusion

Our findings indicate that OLP, OLL and generalised stomatitis are associated with xerostomia. However, xerostomia was not associated with reduction in the salivary flow rates, the number of diseases or number of medications taken on a daily basis, age or gender. There were no differences in UWS, SWS and SPS flow rates between the patients with OLP/OLL and generalised stomatitis and healthy control subjects. On the other hand, the salivary concentrations of total protein and the levels of sIgA were higher in the patient group than in the healthy control group even though the difference was not significant. However, these findings do not aid in the discrimination between OLP and OLL and these conditions with or without contact allergic reactions.

## Abbreviations

ESCD: European Society of Contact Dermatitis
IgG: Immunoglobulin G
OLL: Oral lichenoid lesions
OLP: Oral lichen planus
sIgA: Secretory immunoglobulin A
SPS: Citric acid stimulated parotid saliva
SWS: Chewing stimulated whole saliva
TSH: Thyroid stimulating hormone
UWS: Unstimulated whole saliva
